# Supplementary material for: Atomic structure and plasticity of the CTX-MthK complex investigated by cryo-EM, NMR, and MD simulations
Source: Nat Commun. 2026 Aug 20;17:8724. doi: 10.1038/s41467-026-76925-2 (PMC13493962; doi:10.1038/s41467-026-76925-2)
Supplement: Supplementary file 1 [file 41467_2026_76925_MOESM1_ESM.pdf]

Supporting Information for:

Atomic structure and plasticity of the CTX-MthK complex investigated by cryo-EM, NMR, and MD simulations

Denis Qoraj[1#], Swantje Mohr[1#], Yessenbek K. Aldakul[2,3], Thiemo Sprink[4,5], Carl Öster[1], Taoran Xiao[1], Peter Schmieder[6], Sascha Lange[1], Tillmann Utesch[2], Daniel Roderer[7], Shanshuang Chen[8], Han Sun[2,3], and Adam Lange[1,9*]

[1]Research Unit Molecular Biophysics, Leibniz-Forschungsinstitut für Molekulare Pharmakologie (FMP), Berlin, Germany.
[2]Research Unit Structural Chemistry and Computational Biophysics, Leibniz-Forschungsinstitut für Molekulare Pharmakologie (FMP), Berlin, Germany.
[3]Institute of Chemistry, Technische Universität Berlin, Berlin, Germany.
[4]Core Facility for Cryo Electron Microscopy of the Charité—Universitätsmedizin Berlin at the Max Delbrück Center, Berlin, Germany.
[5]Max Delbrück Center for Molecular Medicine, Technology Platform Cryo-EM, Berlin, Germany.
[6]Core Facility for NMR Spectroscopy, Leibniz-Forschungsinstitut für Molekulare Pharmakologie (FMP), Berlin, Germany.
[7]Leibniz-Forschungsinstitut für Molekulare Pharmakologie (FMP), Berlin, Germany.
[8]Shanghai Institute of Precision Medicine, Ninth People's Hospital, Shanghai Jiao Tong University School of Medicine, Shanghai, China.
[9]Institut für Biologie, Humboldt-Universität zu Berlin, Germany.

*to whom correspondence should be addressed: alange@fmp-berlin.de (A.L.)

#these authors contributed equally.

Cryo-EM data collection, refinement, and validation statistics

Table S1. Cryo-EM data collection, refinement, and validation statistics.

	MthK-CTX (C4, cryoSPARC)	MthK-CTX (C1) (EMD-56657) (PDB 28NO)
**Data collection and processing**		
Magnification	105000	105000
Voltage (kV)	300	300
Electron exposure (e^{-}/Å^2)	60.76	60.76
Defocus range (μm)	-0.6 to 2.6	-0.6 to 2.6
Pixel size (Å)	0.425 (super-resolution)	0.425 (super-resolution)
Symmetry imposed	C4	C1
Initial particle images (no.)	3,502,957	3,502,957
Final particle images (no.)	106,517	92,302
Map resolution (Å)	3.2	4.1
FSC threshold	0.143	0.143
Map resolution range (Å)	1.8-9.2	3.8-6.5
**Refinement**		
Initial model used (PDB code)		5BKI, 2CRD
Model resolution (Å)		3.7
FSC threshold		0.143
Model resolution range (Å)		
Map sharpening *B* factor (Å^2)	109.3	-159.294
Model composition		
Non-hydrogen atoms		3084
Protein residues		393
Ligands		K:1
B factors (Å^2)		
Protein		53.65
Ligand		18.46

R.m.s. deviations	
Bond lengths (Å)	0.003
Bond angles (°)	0.624
Validation	
MolProbity score	1.03
Clashscore	0.96
Poor rotamers (%)	0.3
Ramachandran plot	
Favored (%)	96.34
Allowed (%)	3.66
Disallowed (%)	0.0

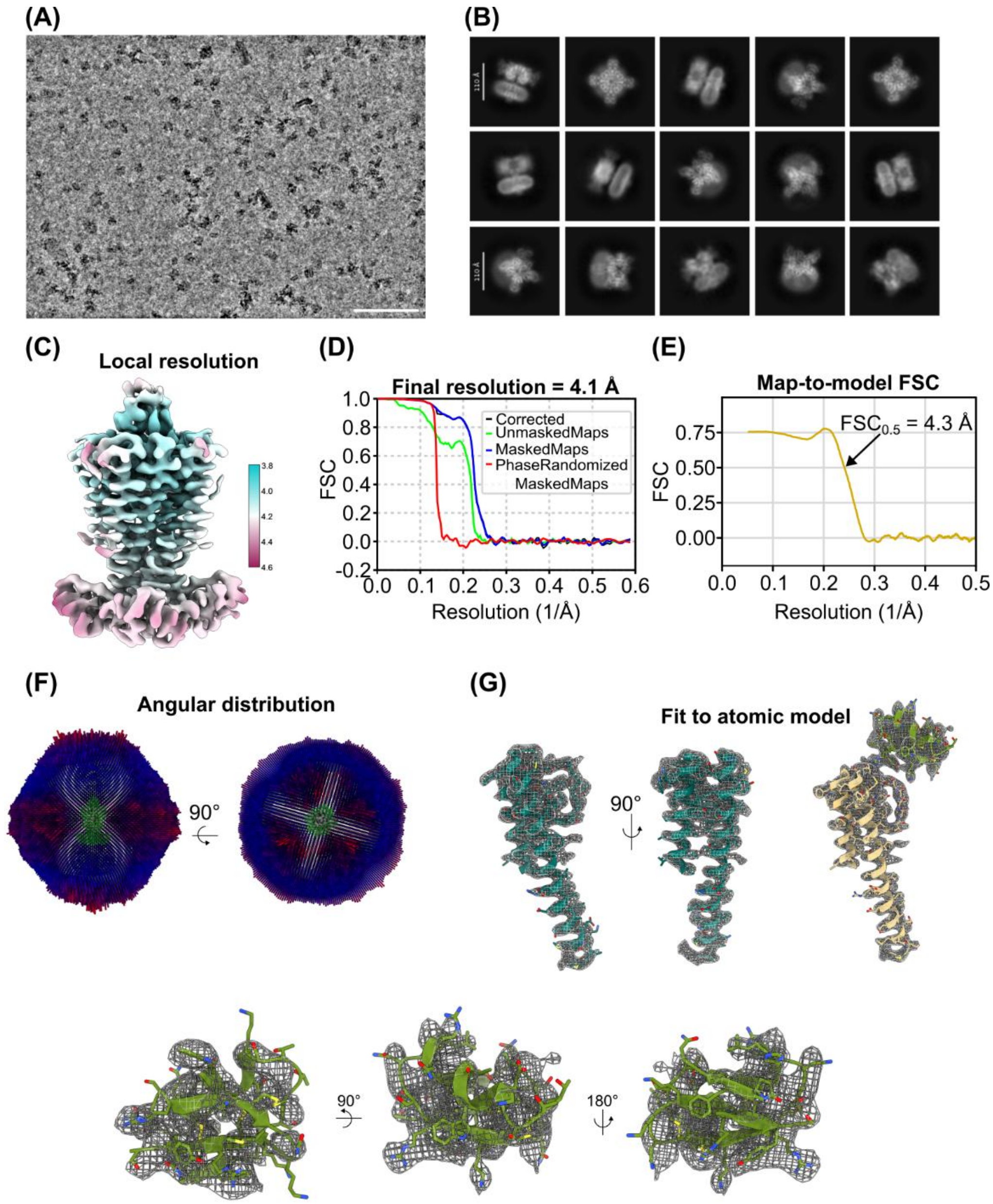

Figure S1. (A) Representative cryo-EM micrograph recorded at 300 kV. Scale bar: 100 nm. **(B)** Representative 2D class averages, showing the MthK-CTX complex in lipid nanodiscs from different views. Scale bar: 110 Å. **(C)** Density map colored by local resolution of the final cryo-EM density map of the MthK/CTX complex from 92,302 particles with C1 applied. **(D)** Map-to-model correlation of the MthK/CTX model against the final unsharpened map. **(E)** Fourier shell correlation (FSC) of the final asymmetric MthK-CTX complex. **(F)** Angular distribution of the final particles. **(G)** Fit of the atomic model in selected parts of the density map. Shown is the final post-processed map.

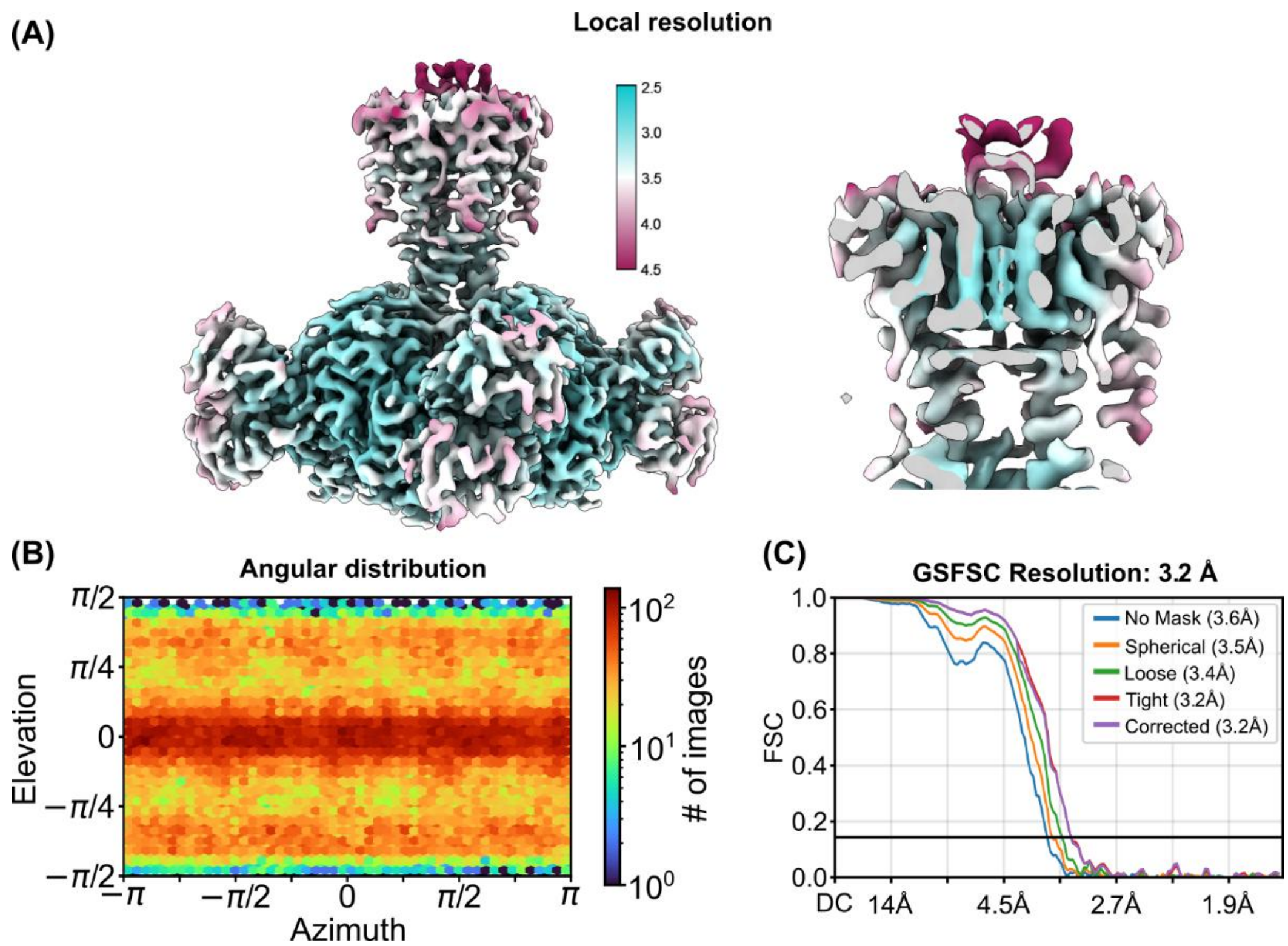

Figure S2. (A) Density map colored by local resolution of the full-length MthK-CTX complex from 106,517 particles with C4 applied. **(B)** Angular distribution of the final particles. **(C)** Fourier shell correlation (FSC) of the full-length MthK-CTX complex.

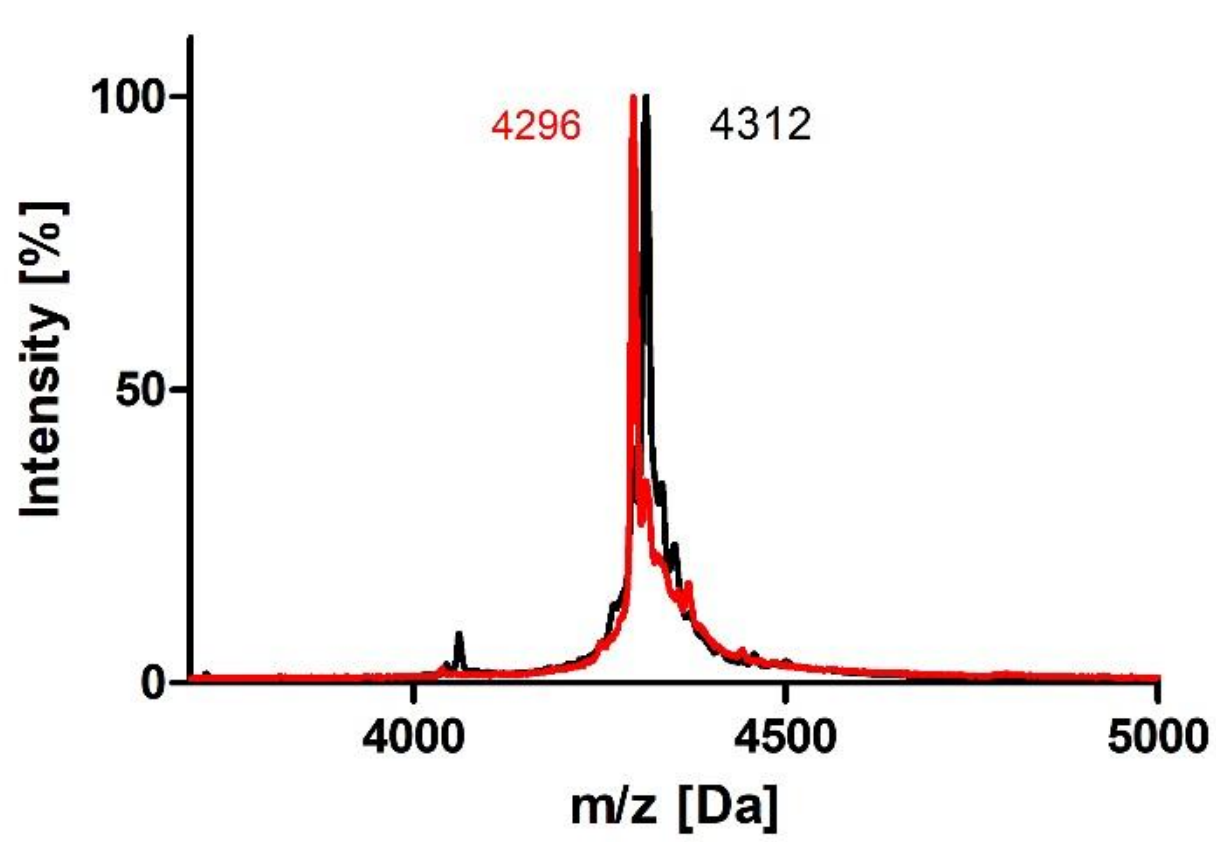

Figure S3. MALDI-TOF MS data of the recombinantly expressed and purified CTX with (red) and without (black) acid-catalyzed cyclization of the N-terminal glutamine to pyroglutamate. The theoretical mass of CTX is 4319 Da. The formation of disulfide bridges (2 Da loss per bond) and cyclisation of the N-terminal glutamine to pyroglutamate (loss of 17 Da) lead to a mass reduction of ~23 Da. It can be seen that even without the native pyroglutamate, disulfide bridges are formed, indicating a native fold for both variants.

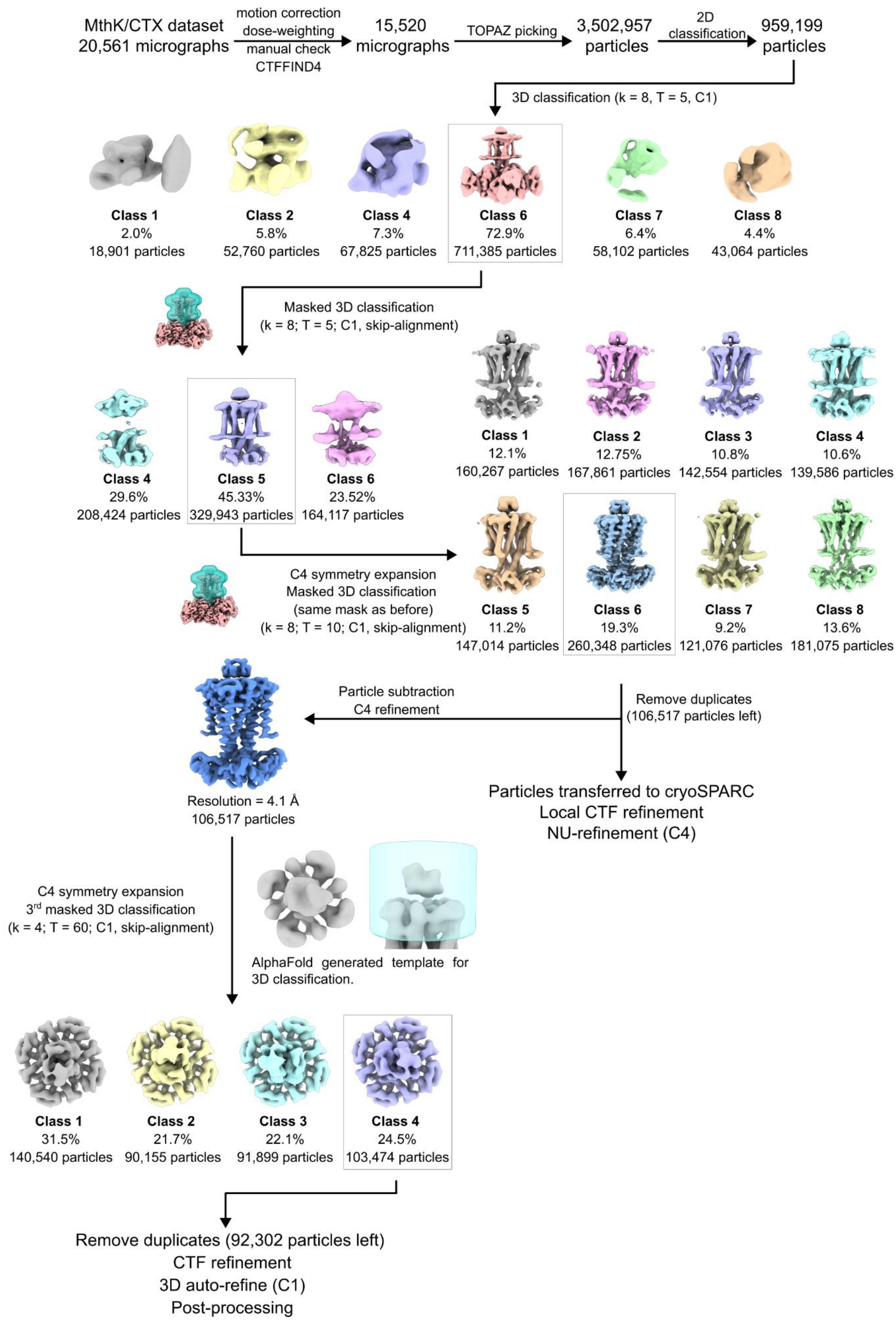

MthK/CTX dataset
20,561 micrographs
motion correction
dose-weighting
manual check
CTFFIND4
15,520
micrographs
TOPAZ picking
3,502,957
particles
2D
classification
959,199
particles
3D classification (k = 8, T = 5, C1)
Class 1
2.0%
18,901 particles
Class 2
5.8%
52,760 particles
Class 4
7.3%
67,825 particles
Class 6
72.9%
711,385 particles
Class 7
6.4%
58,102 particles
Class 8
4.4%
43,064 particles
Masked 3D classification
(k = 8; T = 5; C1, skip-alignment)
Class 4
29.6%
208,424 particles
Class 5
45.33%
329,943 particles
Class 6
23.52%
164,117 particles
Class 1
12.1%
160,267 particles
Class 2
12.75%
167,861 particles
Class 3
10.8%
142,554 particles
Class 4
10.6%
139,586 particles
C4 symmetry expansion
Masked 3D classification
(same mask as before)
(k = 8; T = 10; C1, skip-alignment)
Class 5
11.2%
147,014 particles
Class 6
19.3%
260,348 particles
Class 7
9.2%
121,076 particles
Class 8
13.6%
181,075 particles
Particle subtraction
C4 refinement
Remove duplicates
(106,517 particles left)
Resolution = 4.1 Å
106,517 particles
Particles transferred to cryoSPARC
Local CTF refinement
NU-refinement (C4)
C4 symmetry expansion
3rd masked 3D classification
(k = 4; T = 60; C1, skip-alignment)
AlphaFold generated template for 3D classification.
Class 1
31.5%
140,540 particles
Class 2
21.7%
90,155 particles
Class 3
22.1%
91,899 particles
Class 4
24.5%
103,474 particles
Remove duplicates (92,302 particles left)
CTF refinement
3D auto-refine (C1)
Post-processing

Figure S4. SPA data processing scheme applied for the full-length and truncated MthK-CTX complex. Preprocessing (motion correction and dose-weighting) was performed in cryoSPARC. All subsequent steps were carried out in Relion-5, except for the non-uniform refinement of the full-length MthK-CTX complex (C4), which was performed in cryoSPARC.

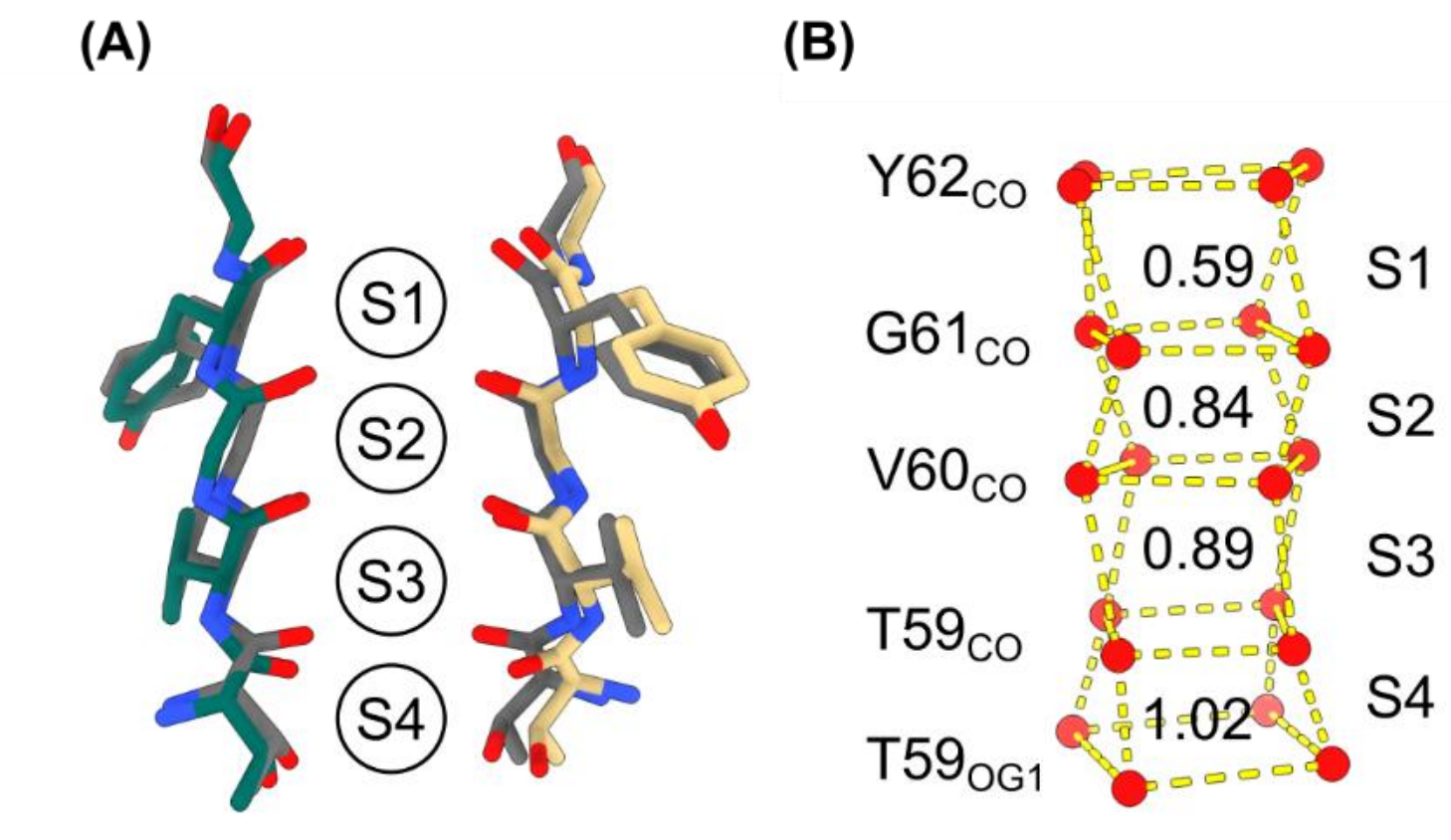

(C)

Binding Site	RMSD (Å) 3LDC	28NO
S1	0.2	0.59
S2	0.36	0.84
S3	0.38	0.89
S4	0.84	1.02

Figure S5. Evaluation of the conductive state of the selectivity filter in the MthK-CTX complex model. **(A)** Alignment of MthK bound to CTX (PDB ID 28NO, green and beige) and the crystal structure of the open conformation of the MthK pore domain (PDB ID: 3LDC, dark gray). For visualization only two chains of both models are shown. **(B)** Measured RMSD values of the selectivity filter CO/OG1 atoms to the aligned water shell oxygens of hydrated potassium ions using the algorithm siter[38]. The oxygen atoms are shown as red spheres and the measured RMSD values are written in the respective ion binding sites in Å. **(C)** Comparison of the measured RMSD values of the selectivity filter CO/OG1 atoms of 3LDC and 28NO to aligned water shell oxygens using siter[38].

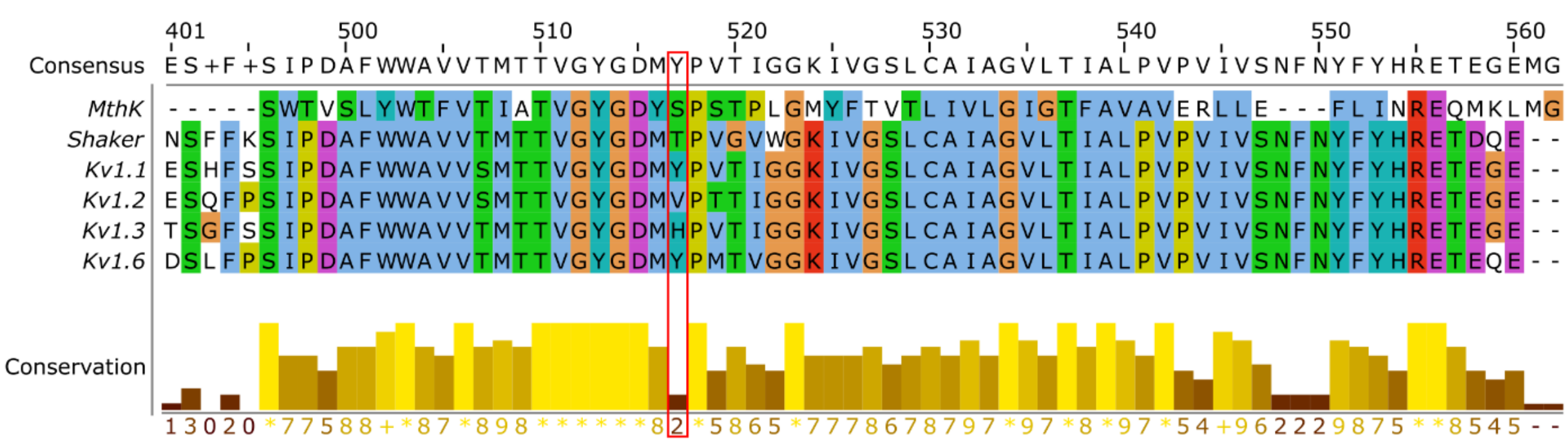

Figure S6. Multiple sequence alignment of MthK, Shaker, and human Kv channels. Shown is the area around the conserved selectivity filter (TVGYG). The residue numbering is based on the consensus sequence. Residues are colored according to the Clustal X colour scheme**.** The red box marks the proposed interaction site of M29 from CTX.

Table S2. Assigned chemical shifts of ^{15}N-labeled CTX in sample buffer acquired with solution NMR. Equivalent protons are marked with an asterisk (*). The chemical shift assignments were deposited in the BMRB (accession code: 53511).

		Chemical shift / ppm						
**Residue Nr.**	**Residue**	**HN**	**N**	**HA**	**HB1**	**HB2**	**HG***	**additional**
3	THR	8.06	113.32	4.97	4.40		1.31	
4	ASN	8.60	119.78					
5	VAL	8.30	123.46	4.03	1.90		1.07	
6	SER	8.69	124.08	5.15	4.01			
7	CYS	8.08	116.46	4.93	3.18	2.98		
8	THR	9.67	112.47	4.84	4.53		1.29	
9	THR	8.36	114.15	4.93	4.51		1.29	
10	SER	8.73	123.52					
11	LYS							
12	GLN	7.37	117.40	4.14				
13	CYS	7.61	113.79	4.78	3.04	2.88		
14	TRP	7.87	125.22	4.61	3.56			HE 10.02
15	SER							
16	VAL	7.17	123.03	3.76	2.23		1.26	1.03
17	CYS	8.62	117.51	4.52	3.11			
18	GLU	8.06	124.41	3.89	2.18		2.40	HE1 7.36<br>HE2 6.70<br>NE 111.52
19	ARG	7.83	118.70	4.21	2.03		1.77	HD* 3.32
20	LEU	8.48	116.30	4.21	1.69			HD* 0.92
21	HIS	7.99	113.82	5.03	2.75	3.53		
22	ASN	7.86	116.88	4.82	3.25	2.78		

23	THR	7.46	110.98	4.86	4.24		1.11	
24	SER	8.29	117.85	4.81	3.88			
25	ARG	7.94	121.22	4.34	1.89		1.56	HD* 3.09
26	GLY	7.62	108.69	3.86	5.23			
27	LYS	9.37	118.64	4.85	1.92		1.48	HD* 2.96
28	CYS	8.85	125.07	4.85	2.76			
29	MET	9.00	130.49	4.89	2.20	1.92	2.55	
30	ASN							
31	LYS	8.65	107.16	3.96	2.27		1.47	HD* 1.82<br>HE* 3.11
32	LYS	7.75	119.32	5.40	1.87		1.50	HE* 3.09
33	CYS	8.60	118.19	5.25	2.68	2.99		
34	ARG	9.68	126.94	5.00	1.76		1.14	HD* 2.74
35	CYS	8.83	123.98	5.58	2.52	3.13		
36	TYR	8.38	122.79	4.91	2.70	3.12		
37	SER	8.23	123.35					

Table S3. Assigned chemical shifts of ^{13}C,^{15}N labeled and deuterated MthK pore domain in K^{+}- and Ca^{2+}-containing buffer, obtained from solid-state NMR experiments with and without unlabeled CTX. Assignments obtained from experiments on CTX-washed-in and NH_4Cl buffer samples are marked with * and **, respectively.

			With CTX	Without CTX
**Residue Nr.**	**Residue**	**Atom**	**Chemical shift / ppm**	**Chemical shift / ppm**
41	ILE	C	176.66	176.79
41	ILE	CA		64.07
41	ILE	H		
41	ILE	N		
42	GLU	C	177.57	177.74
42	GLU	CA	54.94	55.04
42	GLU	H	7.20	7.21
42	GLU	N	114.51	114.30
43	GLY	C	174.81	174.78
43	GLY	CA	45.94	46.00
43	GLY	H	6.61	6.67
43	GLY	N	106.09	106.16
44	GLU	C	176.02	176.12
44	GLU	CA	53.84	53.97
44	GLU	H	5.84	5.84
44	GLU	N	116.08	116.08
45	SER	C	175.64	175.69
45	SER	CA	57.31	57.41
45	SER	H	9.19	9.34
45	SER	N	117.67	117.99
46	TRP	C	177.74	177.84
46	TRP	CA	61.41	61.42

46	TRP	H	9.29	9.27
46	TRP	N	125.14	125.12
47	THR	C	175.85	175.78
47	THR	CA	67.09	67.33
47	THR	H	8.58	8.68
47	THR	N	114.12	114.71
48	VAL	C		177.46
48	VAL	CA	65.93	66.03
48	VAL	H	7.59	7.60
48	VAL	N	120.92	120.70
59	THR	C	172.30**	171.75
59	THR	CA		
59	THR	CB	68.50**	67.96
59	THR	H	-	-
59	THR	N	-	-
60	VAL	C	176.12**	176.63
60	VAL	CA	65.92**	66.09
60	VAL	H	-	-
60	VAL	N	-	-
61	GLY	C	173.63	173.25
61	GLY	CA	47.61**	46.75
61	GLY	H	7.25*	6.99
61	GLY	N	101.56*	99.61
62	TYR	C	177.14	178.58
62	TYR	CA	59.01	59.19
62	TYR	H	5.73	5.69

62	TYR	N	112.12	110.08
63	GLY	C	177.62	175.21
63	GLY	CA	45.77	44.69
63	GLY	H	9.36	9.64
63	GLY	N	99.18	100.88
64	ASP	C		175.54
64	ASP	CA	55.30	55.21
64	ASP	H	8.43	9.47
64	ASP	N	122.70	120.75
65	TYR	C	174.26	174.22
65	TYR	CA	56.93	57.32
65	TYR	H	7.49	7.35
65	TYR	N	115.65	115.57
66	SER	C		169.78
66	SER	CA	56.15	56.60
66	SER	H	8.02	8.42
66	SER	N	114.69	115.40
67	PRO	C	175.82	175.86
67	PRO	CA		62.28
67	PRO	H		
67	PRO	N		
68	SER	C	174.37	174.64
68	SER	CA	58.02	58.07
68	SER	H	9.71	9.79
68	SER	N	116.19	115.85
69	THR	C		173.20

69	THR	CA	56.73	58.65
69	THR	H	8.39	8.57
69	THR	N	115.75	115.31
70	PRO	C	178.19	178.25
70	PRO	CA		65.69
70	PRO	H		
70	PRO	N		
71	LEU	C	178.59	178.59
71	LEU	CA	57.98	58.08
71	LEU	H	8.52	8.62
71	LEU	N	116.20	116.42
72	GLY	C	177.86	177.90
72	GLY	CA	46.74	46.91
72	GLY	H	8.04	8.14
72	GLY	N	106.46	106.82
73	MET	C	177.50	177.77
73	MET	CA	60.46	60.66
73	MET	H	8.64	8.75
73	MET	N	126.69	126.71
74	TYR	C		
74	TYR	CA	61.97	62.80
74	TYR	H	8.31	8.37
74	TYR	N	117.88	118.18

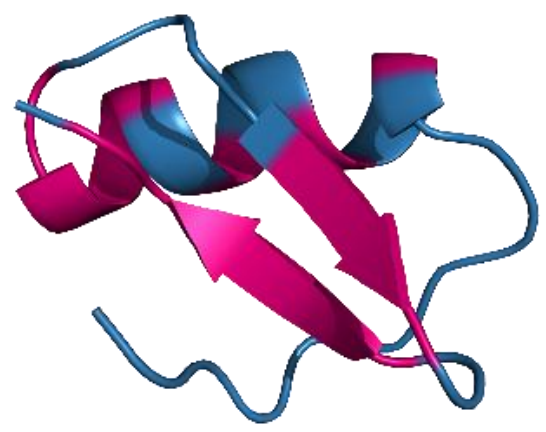

Figure S7. TALOS+ prediction mapped on the atomic CTX structure (PDB ID: 2CRD). Pink residues match the secondary structure prediction. Blue residues are predicted to be coil regions or couldn't be unambiguously assigned.

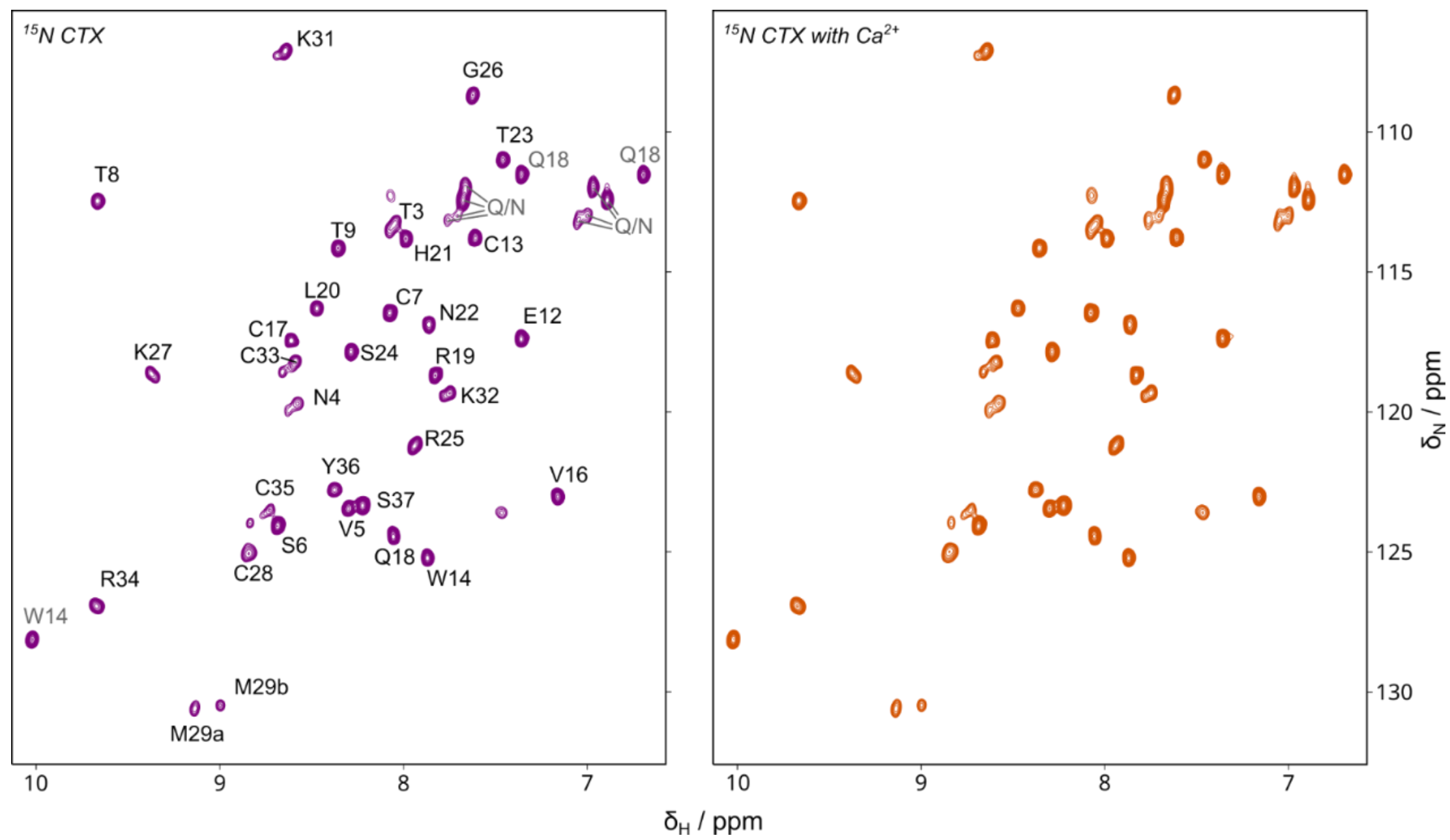

Figure S8. ^{15}N-^{1}H HSQC solution NMR spectra of free CTX in buffer without (left) and with (right) Ca^{2+}. Assignments are marked in the left spectrum. The spectra were recorded at 600 MHz at a temperature of 300 K.

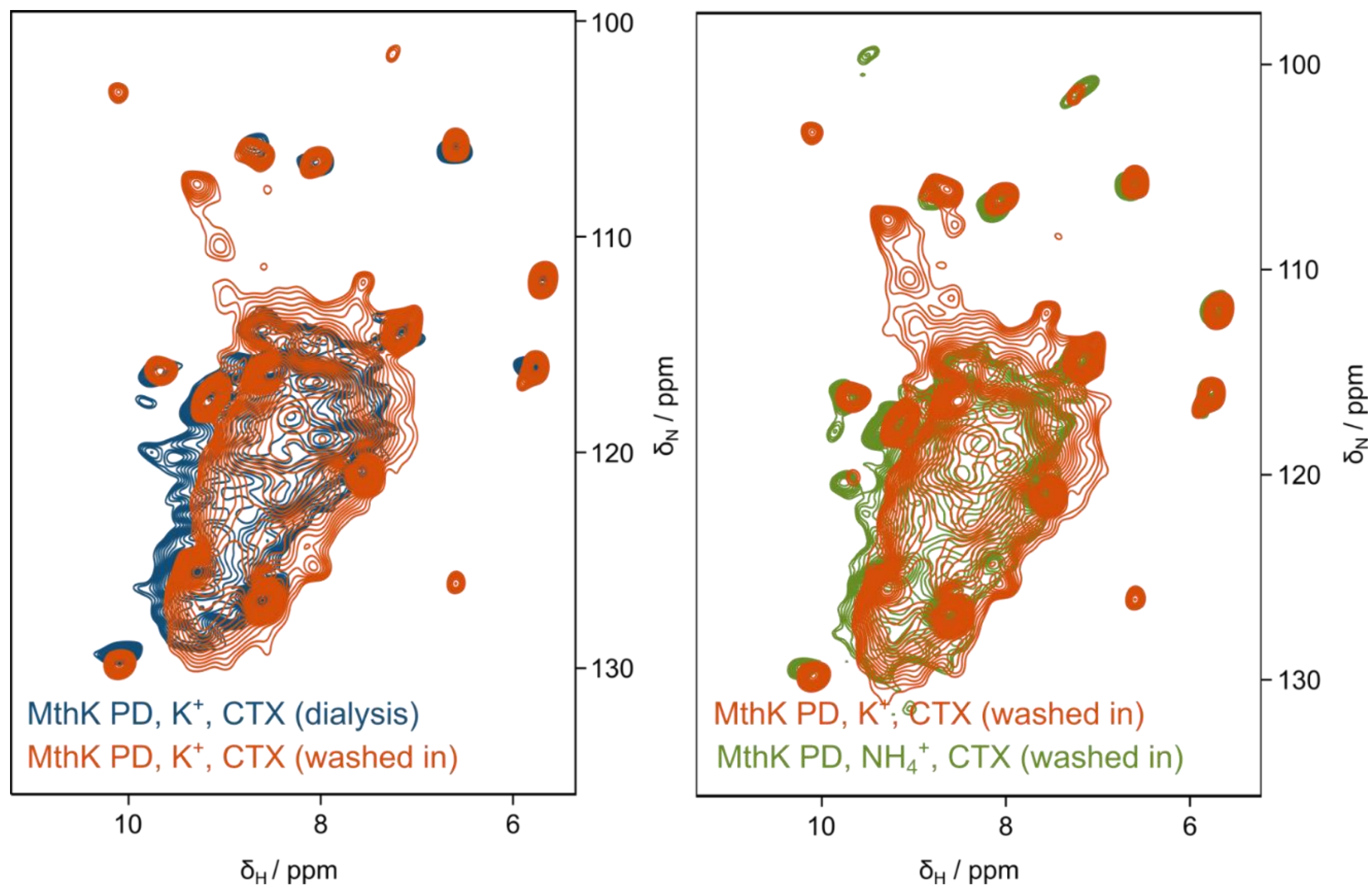

Figure S9. (H)NH spectra of MthK-PD. MthK-PD in 100 mM potassium buffer is shown with CTX added during dialysis (blue) and washed in after reconstitution (red). In green, MthK-PD in $^{15}NH_4^+$-buffer and with CTX washed in is shown.

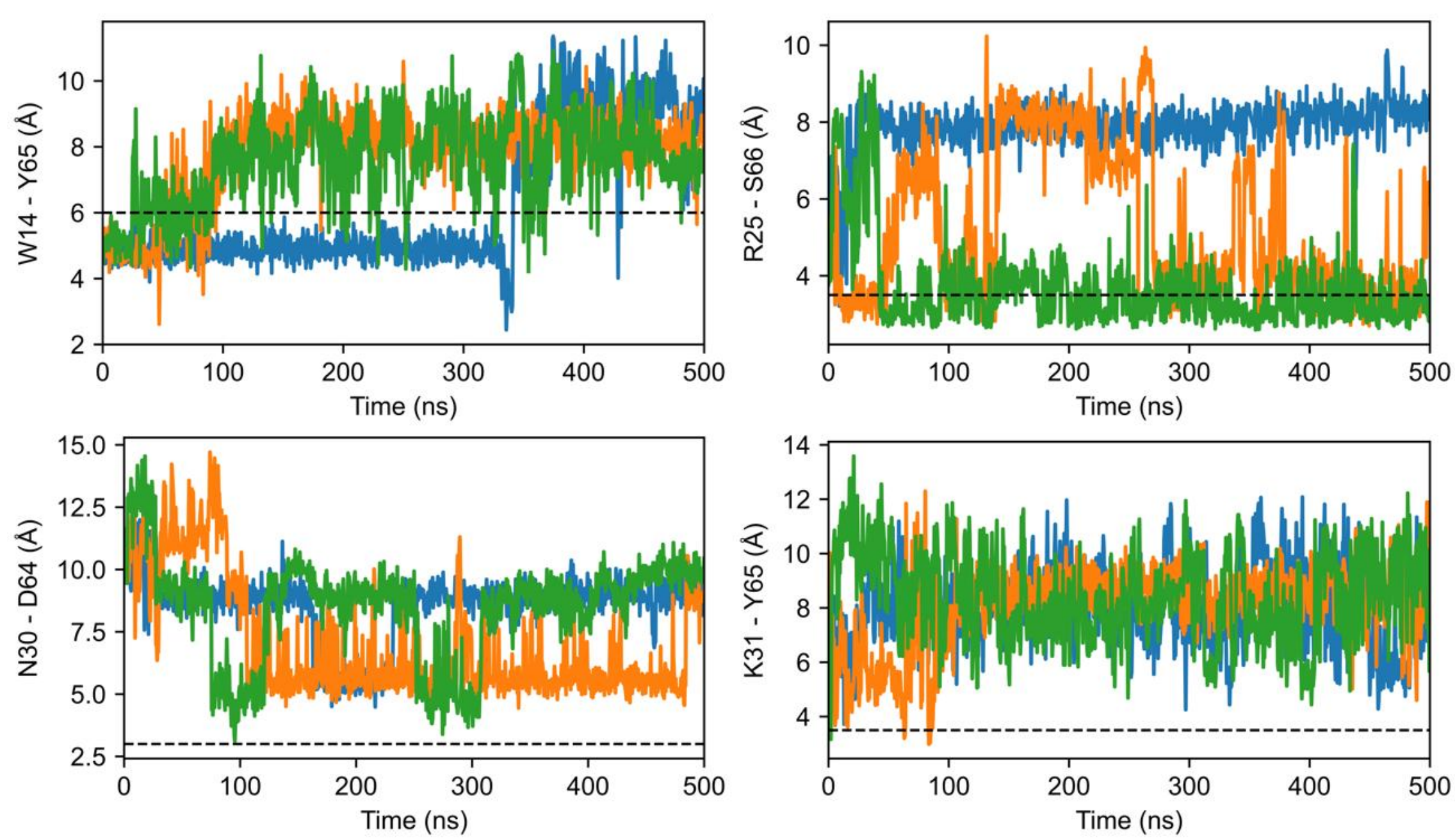

Figure S10. Minimum distances between CTX (W14, R25, N30, K31) and MthK (Y65, S66, D64). Each line corresponds to a single MD simulation and dashed black lines indicate the distances in the cryo-EM model.

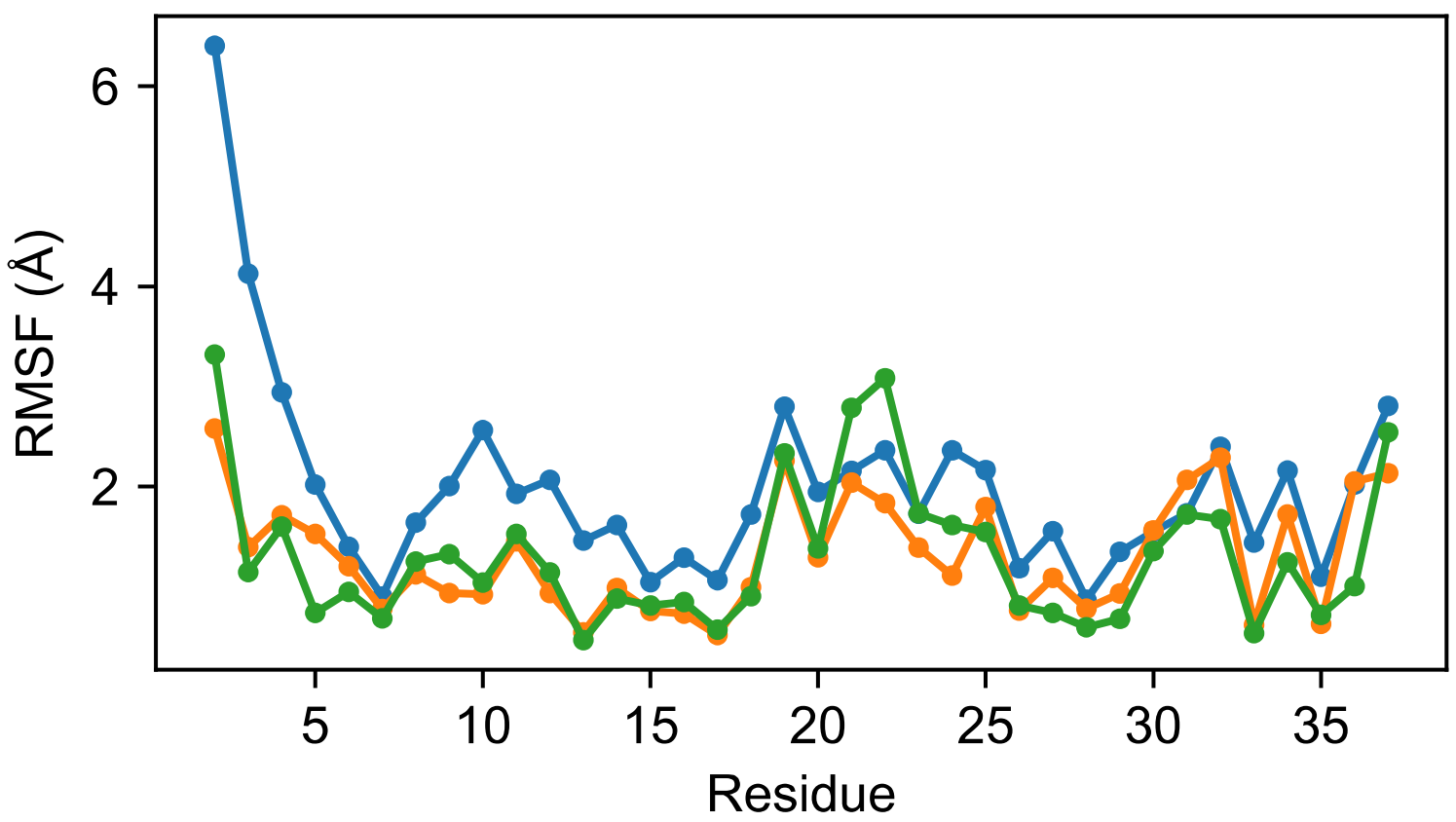

Figure S11. Root-mean-square fluctuation of the CTX M29I mutant from three 500 ns MD simulations shown in different colors.

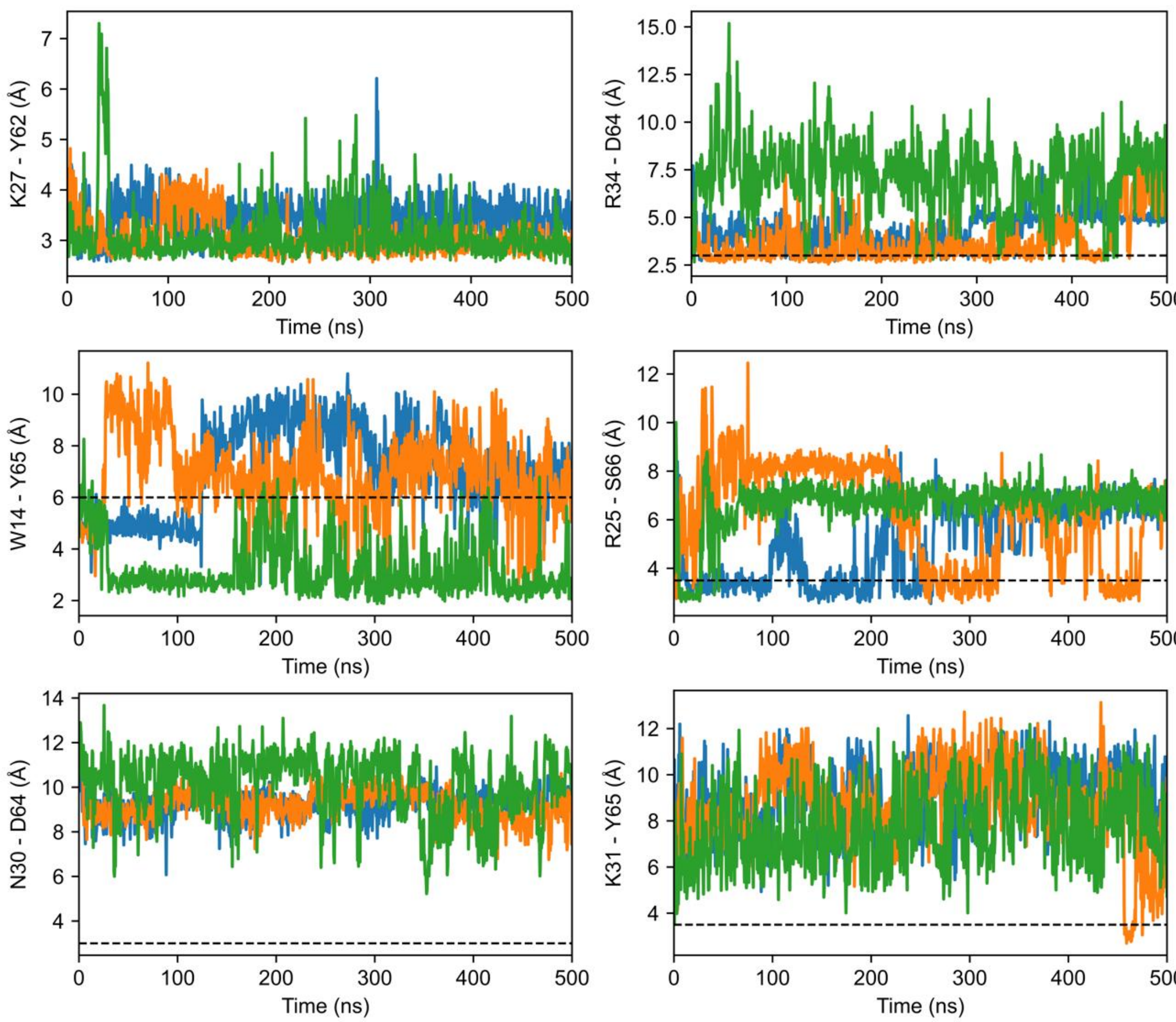

Figure S12. Minimum distances between residues of the CTX M29I mutant (K27, R34, W14, R25, N30, K31) and MthK (Y62, D64, Y65, S66). Each line corresponds to a single MD simulation and dashed black lines indicate the distances in the cryo-EM model.

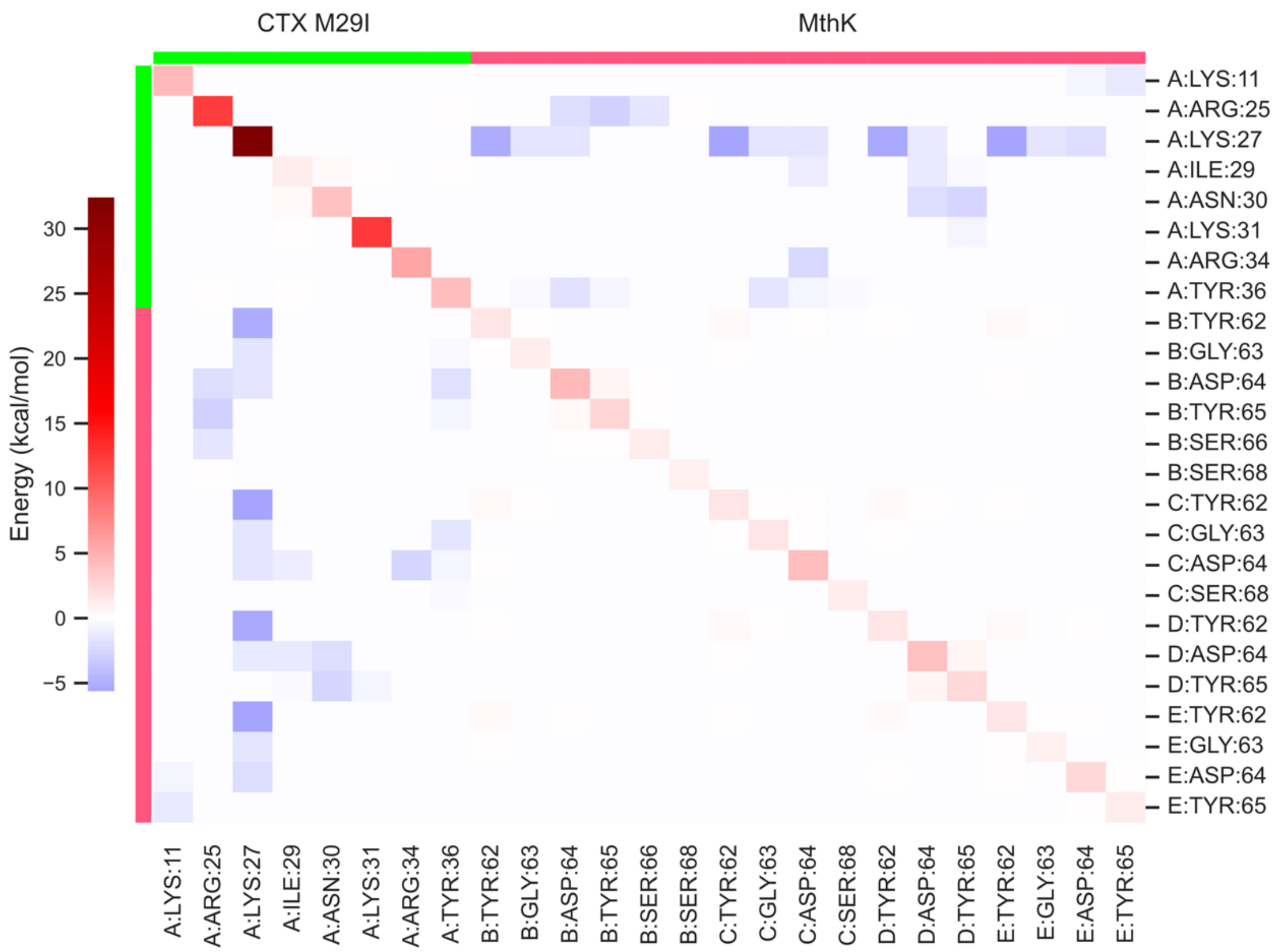

Figure S13. Estimation of the binding free energy of the M29I mutant CTX-MthK complex using the MM/PBSA method. CTX M29I is shown in green and MthK is shown in red.

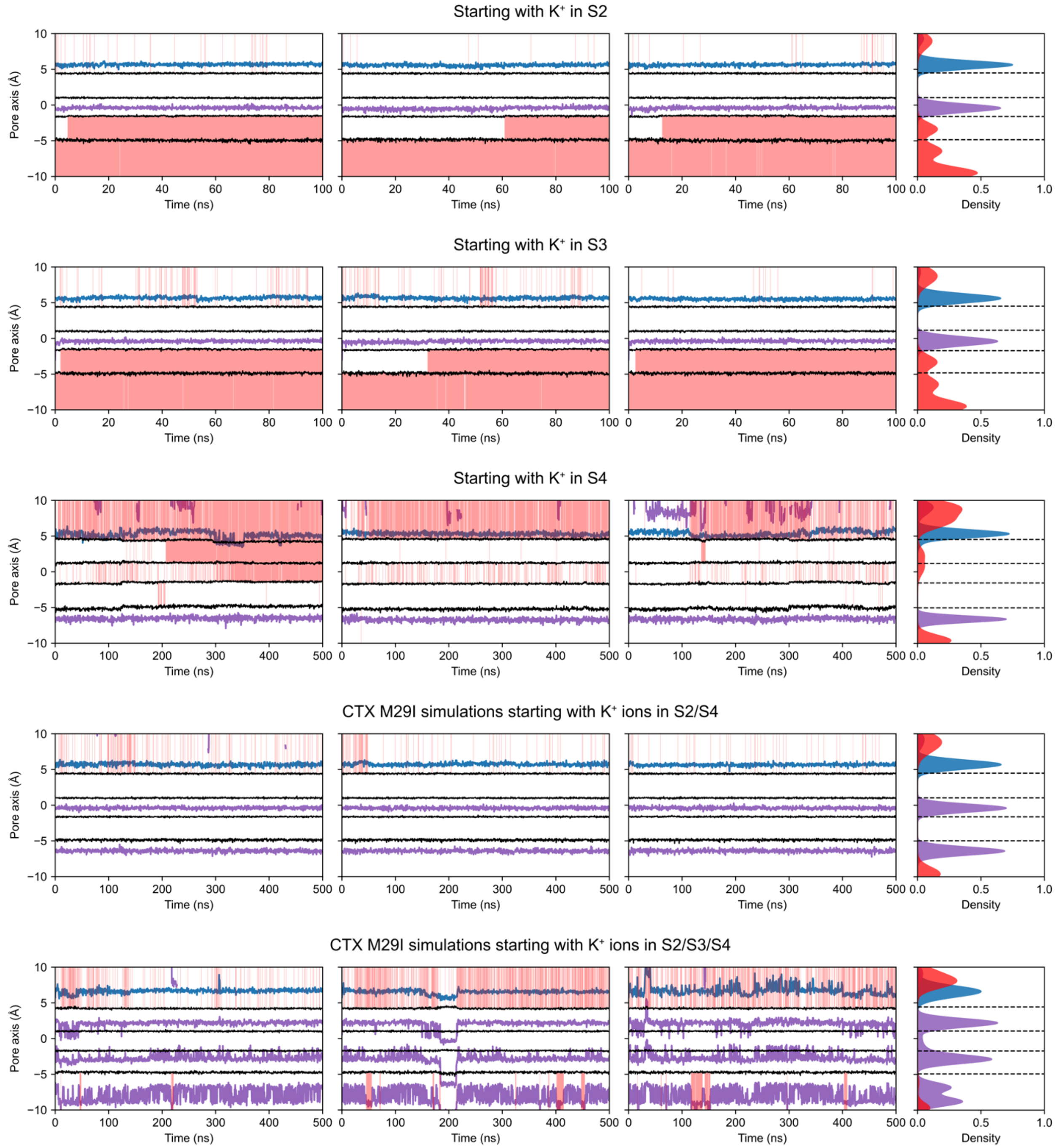

Figure S14. The occupancies of water (red), K27 of CTX (blue), and bound K^+ ions (purple) in the SF binding sites during MD simulations. Black solid and dashed lines indicate the center of geometry of carbonyl oxygen atoms delimiting the SF binding sites.

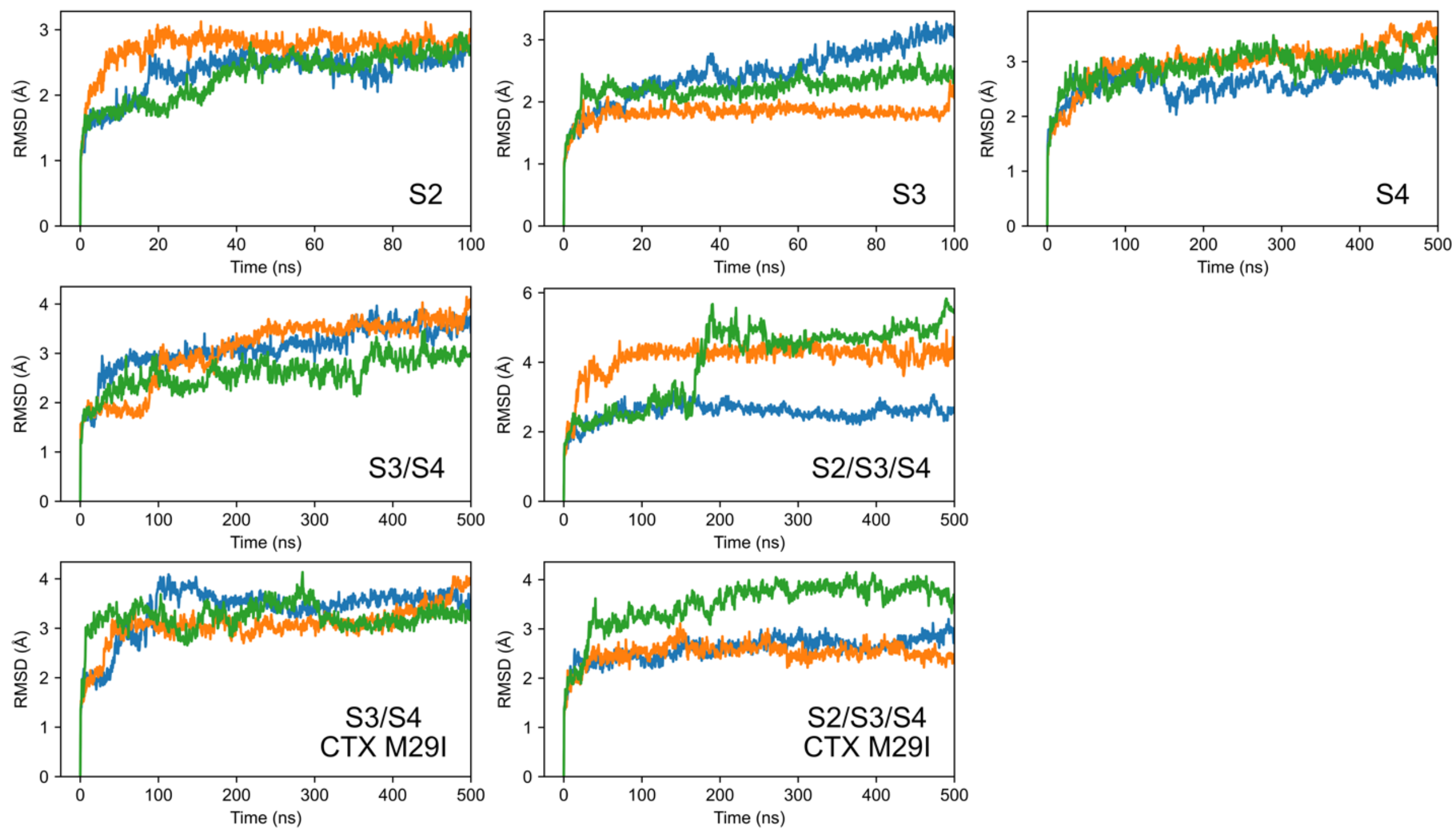

Figure S15. Root-Mean-Square Deviation (RMSD) of the CTX-MthK complex in MD simulations. Each simulation is colored in different color.

Movie S1. Concatenated trajectories from 3x500 ns MD simulations of the CTX-MthK complex with two K^+ ions in the SF.

Movie S2. Concatenated trajectories from 3x500 ns MD simulations of the CTX-MthK complex with three K^+ ions in the SF.

Table S4. MD simulations of the CTX-MthK complex.

System	N of atoms	N of TIP3	N of POPE: POPG	N of K^+	N of Cl^-	Simulation box dimensions (nm x nm x nm)	K^+ ions in the SF	N of runs	Simulation time (ns)
CTX-MthK complex	64428	12664	120:40	64	22	7.8x7.8x10.2	S2/S3	3	500
							S2/S3/S4	3	500
							S2	3	100
							S3	3	100
							S4	3	500
CTX-MthK complex with M29I mutation on toxin	64430	12664	120:40	64	22	7.8x7.8x10.2	S2/S3	3	500
							S2/S3/S4	3	500

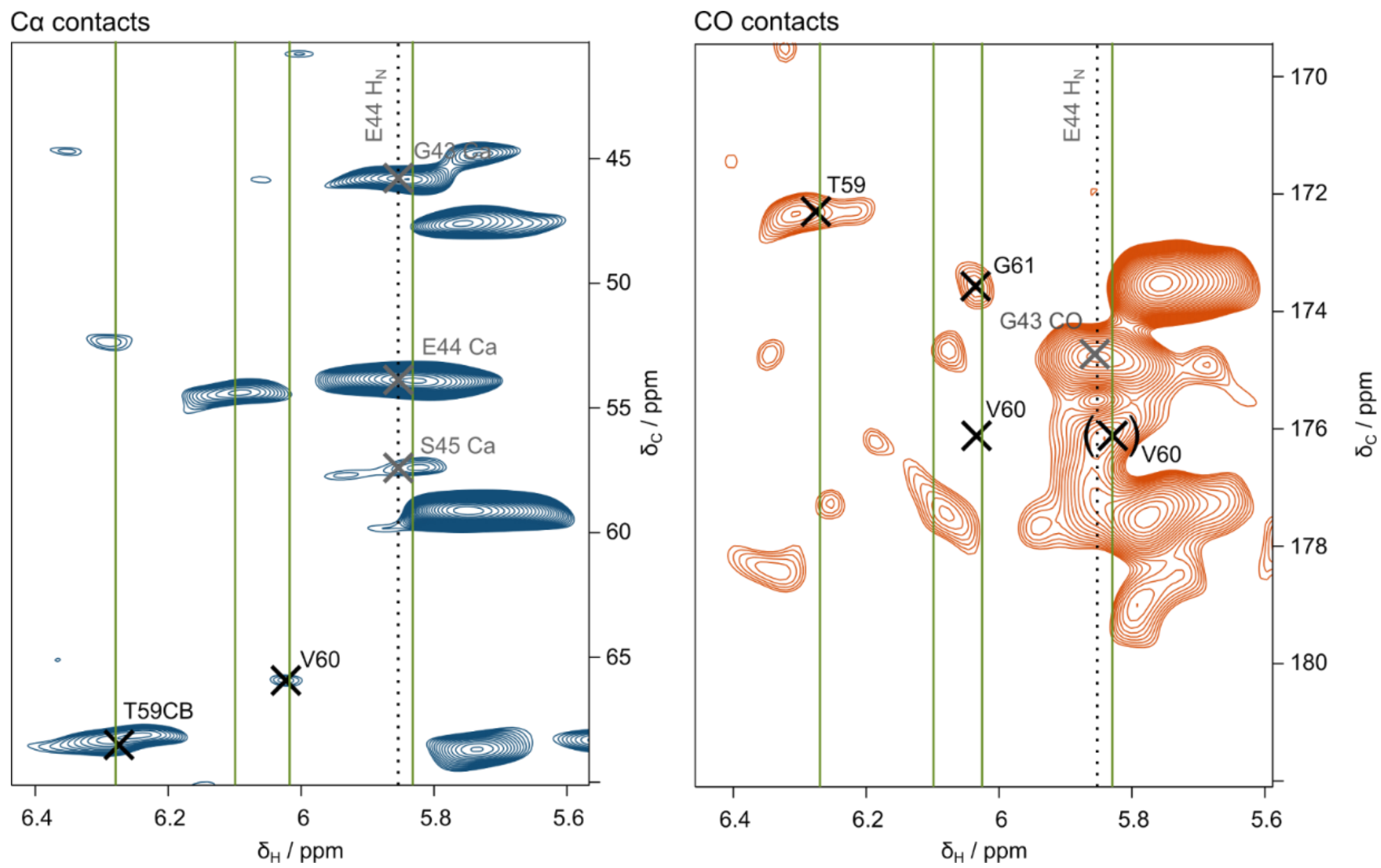

Figure S16. (H)CH CP spectra of MthK-PD with washed in CTX, in $^{15}NH_4Cl$-buffer. Experiments were measured with a 7 ms CP mixing time. Contacts between ammonium ions (H-chemical shift marked with green lines) and C-atoms of selectivity-filter residues are marked. Contacts between the backbone HN of E44 and other carbon atoms are marked in grey.

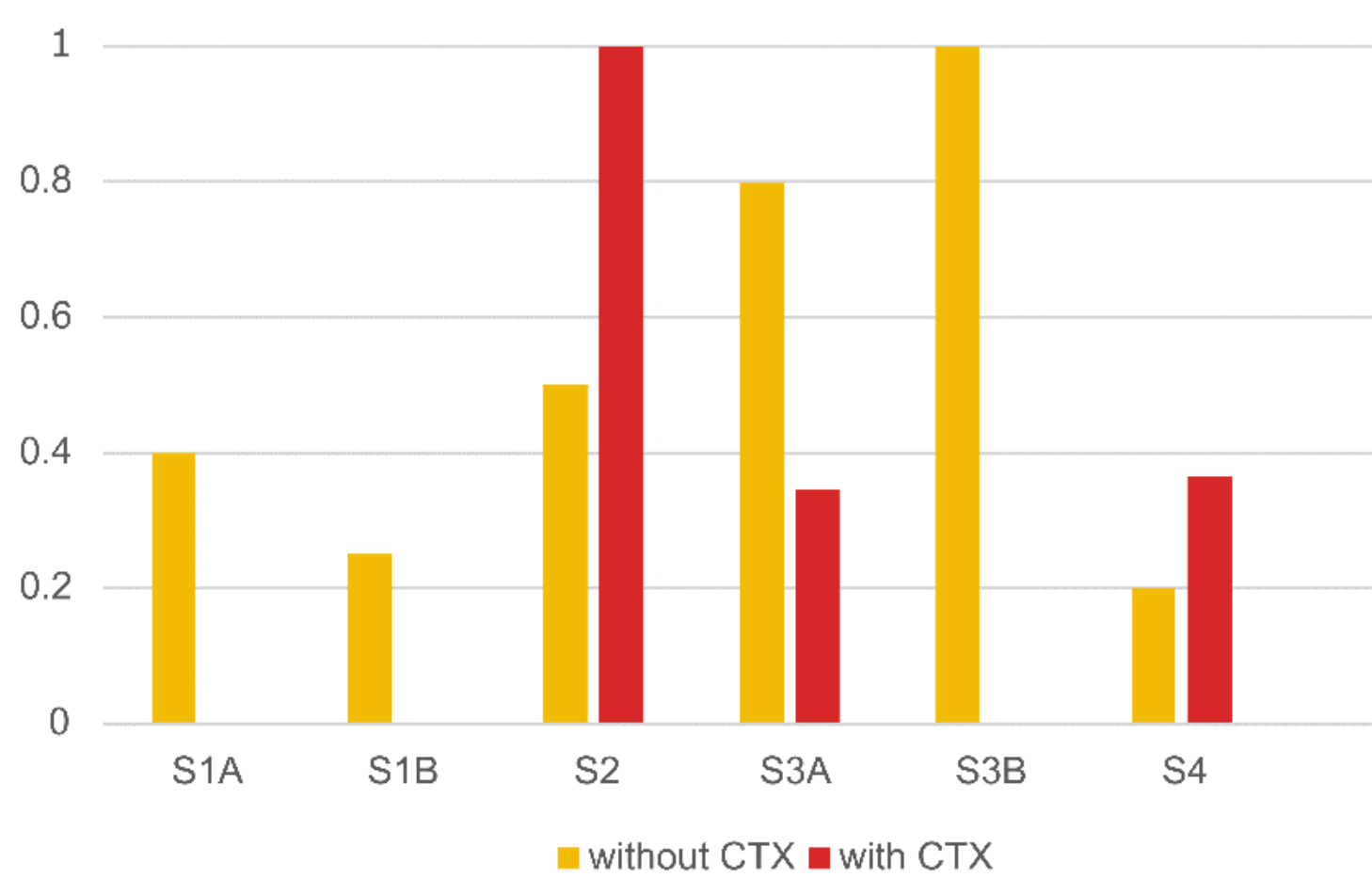

Figure S17. Relative intensities of bound $^{15}NH_4^+$ ions in the selectivity filter of MthK in the free (yellow) and CTX-bound (red) state.
