# Supplementary material for: Atomic structure and plasticity of the CTX-MthK complex investigated by cryo-EM, NMR, and MD simulations
Source: Nat Commun. 2026 Aug 20;17:8724. doi: 10.1038/s41467-026-76925-2 (PMC13493962; doi:10.1038/s41467-026-76925-2)
Supplement: Supplementary file 2 [file 41467_2026_76925_MOESM2_ESM.pdf]

Description of Additional Supplementary Files

File Name: Supplementary Movie 1

Description: Concatenated trajectories from 3x500 ns MD simulations of the CTX-MthK complex with two K+ ions in the SF.

File Name: Supplementary Movie 2

Description: Concatenated trajectories from 3x500 ns MD simulations of the CTX-MthK complex with three K+ ions in the SF.
