# Supplementary material for: Atomic structure and plasticity of the CTX-MthK complex investigated by cryo-EM, NMR, and MD simulations
Source: Nat Commun. 2026 Aug 20;17:8724. doi: 10.1038/s41467-026-76925-2 (PMC13493962; doi:10.1038/s41467-026-76925-2)
Supplement: Supplementary file 4 [file 41467_2026_76925_MOESM6_ESM.pdf]

nature portfolio

# Peer Review File

# Atomic structure and plasticity of the CTX-MthK complex investigated by cryo-EM, NMR, and MD simulations

Corresponding Author: Professor Adam Lange

**This file contains all reviewer reports in order by version, followed by all author rebuttals in order by version.**

Version 0:

Reviewer comments:

Reviewer #1

(Remarks to the Author)
The authors study binding of a scorpion toxin, charybdotoxin (CTX), to a prokaryotic K+ channel MthK using cryo-EM, NMR and MD simulations. The study is comprehensive, the data are of high quality, and the conclusions are supported by the data. My main critique is that the primary conclusions (lysine inserts into SF, altered ion occupancy in SF) are already known from structural and functional studies of other K+ channel/toxin complexes, including CTX bound to a mammalian Kv channel. The authors have referenced these studies (refs 26-31) appropriately. However, I support publication of the manuscript as in my opinion it is a rare example of work that studies the same system using a truly integrative approach.

Comments:
1. The chemical shift perturbations in Fig 3: CTX binds asymmetrically to the tetramer. In the limit of slow exchange, separate peaks should be observed for the different subunits. The authors could clarify this point for the readers that might not be familiar with how to interpret the data.
2. The X-tal structure of CTX bound to Kv1.2 was reported in 2013. The findings at the time seemed to be at odds with the SSNMR study of kaliotoxin bound to KcsA in 2006. The SSNMR study showed clear chemical shift changes in both the channel and the toxin, while the X-tal structure showed almost no conformational changes in the channel and toxin. This study echoes the same point, and it is an important one to re-emphasize for the field: chemical shift changes seen by NMR do not always translate to large scale conformational changes seen by X-tal/cryo-EM. The authors already mention this on pg 8. I would encourage the authors to consider adding this small but important distinction to the discussion if they can find a place for it. Another literature example of this behavior: https://doi.org/10.1038/nature16577.
3. Is it possible to estimate the relative occupancy of NH4+ at different sites in the SF from quantitative (DP) NMR spectra? I am aware this has been done by crystallography but I am not sure if anyone has done it already using NMR in membranes. This would nicely complement the MD work.
4. Related to this, others have measured changes in ion occupancy upon CTX binding in their X-tal/EM data (see the Banerjee 2013 or Sigworth's recent Kv1.2 work). Would be good to discuss briefly.
5. I am a little surprised that the CTX purification does not include a refolding step to ensure the 3 disulfides are formed correctly. Do the bridges stay intact through the purification? Clearly it works, but just for my curiosity..
6. The yellow spectrum is a little difficult to see in Fig2a/3a. Is it possible to make it clearer?

Reviewer #2

(Remarks to the Author)
This manuscript presents a comprehensive structural investigation of the charybdotoxin (CTX)-MthK complex using an integrative approach combining cryo-EM, solution- and solid-state NMR, and molecular dynamics simulations. The technical execution is of high quality, and the combination of multiple complementary methods represents the strength of the work.

Major strengths:
1. The integrative structural biology approach effectively overcomes limitations inherent to any single technique, particularly for characterizing small peptide-protein complexes where symmetry mismatch poses challenges.
2. The solid-state NMR experiments with $^{15}NH_4^+$ ions provide valuable direct evidence for altered ion occupancy in the selectivity filter upon toxin binding, demonstrating occupancy at S2 and S4 while S1 remains vacant.

3. MD simulations reveal important dynamics of the toxin-channel interface, showing that while K27 serves as a stable anchor, other residues exhibit flexibility on the nanosecond timescale.
4. The work provides a well-characterized example of wild-type toxin binding without fusion constructs or heavy-atom derivatives.

Major concerns:
1. Limited conceptual novelty. While technically sound, the study does not provide fundamentally new mechanistic insights beyond what has been established by previous structures of toxin-channel complexes, particularly the Kv1.2–ChTx structure (Banerjee et al., 2013, eLife) and the recent Kv1.2–dendrotoxin structure (Wu et al., 2024, eLife). The general binding mode—with a conserved lysine inserted into the selectivity filter and peripheral electrostatic/hydrophobic contacts—has been well established. The observation of "structural plasticity" and dynamic binding, while nicely demonstrated here, has also been inferred from previous computational and biophysical studies.
2. Selectivity filter conformation. The authors report that CTX binding does not induce structural rearrangement of the selectivity filter itself, only changes in ion occupancy. This conclusion would benefit from comparison with recent comprehensive analyses of selectivity filter conformations across $K^+$ channels, including during C-type inactivation. For instance, Scherbakov et al. (10.1016/j.ijbiomac.2025.140690) systematically examined SF structures and conformational transitions. Such comparison would strengthen the authors' interpretation that the filter remains in a "non-collapsed" conformation despite altered ion occupancy.
3. Missed opportunity regarding toxin specificity determinants. The authors identify "structural plasticity" as the basis for CTX's broad channel promiscuity, with K27 as an anchor and flexible peripheral contacts. However, they do not address a critical unresolved question in the field: the M29I mutation in CTX causes a dramatic 1500-fold change in affinity for Kv1.2 (10.1134/S002209302206031X, 10.1016/j.toxicon.2023.107181), yet the structural basis for this specificity switch remains unexplained. The authors identify M29 as showing chemical shift perturbations and suggest it coordinates R34 and Y36, but do not discuss how this position could act as an "evolutionary switch" controlling selectivity across the KTX toxin family. Given the authors' integrated structural approach and focus on binding plasticity, investigating the M29I mutant structure would have provided genuine mechanistic novelty and directly addressed an outstanding question in the field.

Minor points:
• Line 393-400: The comparison with the KcsA-Kv1.3-KTX study is somewhat superficial. The authors suggest differences may be system-dependent but do not explore whether their ammonium-based NMR approach could resolve this question.
• The cryo-EM resolution (4.1 Å for asymmetric reconstruction) limits atomic-level interpretation of some CTX–MthK contacts. The authors appropriately acknowledge this but might discuss how future technical advances could improve resolution.

Recommendation:
This is competent structural work with solid technical execution. However, given that the fundamental binding mechanism has been established by previous structures and that the study does not address key unresolved questions (such as the M29I specificity determinant), the conceptual advance appears incremental rather than substantial. The work would be well-suited for a more specialized venue such as Communications Biology, where it would make a valuable contribution to the structural toxinology literature without requiring the broad impact expected for Nature Communications.

Version 1:

Reviewer comments:

Reviewer #1

(Remarks to the Author)
The authors have sufficiently addressed my minor comments and improved the manuscript. I support publication in the current form.

Reviewer #2

(Remarks to the Author)
The authors have substantially addressed all major concerns raised in the previous review. The addition of RMSD comparison and siter-based analysis of the selectivity filter conformation (Concern 2) is quantitatively convincing (RMSD = 0.188 Å vs. open MthK, conductive state confirmed). The new MD simulations of the M29I mutant (Concern 3) represent a genuine experimental addition rather than a textual revision, and the proposed structural rationale for methionine flexibility as an "affinity switch" is mechanistically plausible. All minor points have been resolved, including removal of the superficial KcsA-Kv1.3-KTX comparison and addition of a discussion of future cryo-EM resolution improvements.

One point remains partially open: the MD simulations of M29I on MthK show no change in binding free energy, which is noted but not deeply discussed — likely because MthK is a prokaryotic surrogate and the Kv1.2-specific effect of M29I cannot be fully recapitulated in this system. This limitation should be briefly acknowledged in the Discussion. With this minor clarification, the manuscript is suitable for publication.

**Open Access** This Peer Review File is licensed under a Creative Commons Attribution 4.0 International License, which permits use, sharing, adaptation, distribution and reproduction in any medium or format, as long as you give appropriate credit to the original author(s) and the source, provide a link to the Creative Commons license, and indicate if changes were made.
In cases where reviewers are anonymous, credit should be given to 'Anonymous Referee' and the source.
The images or other third party material in this Peer Review File are included in the article's Creative Commons license, unless indicated otherwise in a credit line to the material. If material is not included in the article's Creative Commons license and your intended use is not permitted by statutory regulation or exceeds the permitted use, you will need to obtain permission directly from the copyright holder.
To view a copy of this license, visit https://creativecommons.org/licenses/by/4.0/

**Reply to reviewer comments:**

Reviewer #1

The authors study binding of a scorpion toxin, charybdotoxin (CTX), to a prokaryotic K+ channel MthK using cryo-EM, NMR and MD simulations. The study is comprehensive, the data are of high quality, and the conclusions are supported by the data. My main critique is that the primary conclusions (lysine inserts into SF, altered ion occupancy in SF) are already known from structural and functional studies of other K+ channel/toxin complexes, including CTX bound to a mammalian Kv channel. The authors have referenced these studies (refs 26-31) appropriately. However, I support publication of the manuscript as in my opinion it is a rare example of work that studies the same system using a truly integrative approach.

We thank the reviewer for their analysis and positive evaluation of our work.

Comments:

1. The chemical shift perturbations in Fig 3: CTX binds asymmetrically to the tetramer. In the limit of slow exchange, separate peaks should be observed for the different subunits. The authors could clarify this point for the readers that might not be familiar with how to interpret the data.

To address this point, we added the following sentences to the manuscript (page 10):

> ”As seen in the cryo-EM reconstruction of the channel-toxin complex, CTX binds asymmetrically to the MthK surface. In the limit of slow exchange (e.g. on a timescale of milliseconds to seconds), this should result in distinct peaks for each subunit of the channel. Interestingly, this cannot be observed in our experiments. While for residues inside the selectivity filter, one could imagine that these differences in electrostatic environment are very small, and peak differences could simply be not resolved, for the upper part of the channel, this should not be the case. Here, motions of the CTX molecule could lead to an averaging of chemical shift changes, investigated in detail in the following section.”

2. The X-tal structure of CTX bound to Kv1.2 was reported in 2013. The findings at the time seemed to be at odds with the SSNMR study of kaliotoxin bound to KcsA in 2006. The SSNMR study showed clear chemical shift changes in both the channel and the toxin, while the X-tal structure showed almost no conformational changes in the channel and toxin. This study echoes the same point, and it is an important one to re-emphasize for the field: chemical shift changes seen by NMR do not always translate to large scale conformational changes seen by X-tal/cryo-EM. The authors already mention this on pg 8. I would encourage the authors to consider adding this small but important distinction to the discussion if they can find a place for it. Another literature example of this behavior: https://doi.org/10.1038/nature16577.

We thank the reviewer for acknowledging this important observation of our study. To point out this finding not only in our results section, but return to it also in the discussion, we added the following sentences into it (page 16):

> "Especially when comparing our study with previous NMR studies, in should be pointed out that when analyzing chemical shift changes in NMR experiments, one must carefully distinguish between those caused by structural rearrangements, and those induced by changes in the chemical environment, for example the establishment or loss of electrostatic interactions or hydrogen bonds, of the respective residues. Methods for describing these interactions, like the analysis of CSPs of different nuclei, as demonstrated in this and several other studies[29,45], or the combination with other structure-determining techniques, can be of major importance in these cases to reach a well-supported conclusion."

In addition to that, we deleted the following sentences from the discussion, as suggested by another reviewer (page 16):

> "In a previous ssNMR investigation on the binding of KTX to the chimeric channel KcsA-Kv1.3, chemical shift changes, e.g., for Y78 Cα (KcsA-Kv1.3) - indicative of backbone torsional angle changes - as well as distance restraints involving Y78 recorded using NHHC experiments[29] pointed towards structural changes in the upper part of the SF. An investigation of this system with the methods employed in the current study may be worthwhile, in particular, the direct detection of ions using the ammonium approach. However, differences observed may also depend on the specific system of interest, as the selectivity filter in different ion channels may respond differently upon toxin binding."

3. Is it possible to estimate the relative occupancy of NH4+ at different sites in the SF from quantitative (DP) NMR spectra? I am aware this has been done by crystallography but I am not sure if anyone has done it already using NMR in membranes. This would nicely complement the MD work.

We thank the reviewer for this suggestion. It is indeed unclear whether the observed peak intensities directly correlate with site occupancies, as the dynamics of the ions are expected to influence the signal intensities in the INEPT spectra. A detailed characterization of these dynamics is challenging due to the inherently low sensitivity of the system. In a previous publication from our group (Öster et al., 2025, https://doi.org/10.1021/jacs.5c16155), peak intensities obtained from INEPT-based spectra have been compared, although under the same restrictions mentioned above. Nevertheless, we calculated relative intensities from the measured CP-spectra, and added them to the SI (SI Fig. 17). Highest intensity can be found for S2, while S3 and S4 show comparable, but significantly lower, intensities. This would fit to our suggestion of a dynamic ion exchange between these two binding sites. We as well added the following sentence to the results (page 14):

> "Although the INEPT magnetization transfer is influenced by the dynamics as well, we additionally compared the intensities of the ammonium ion signals in the free and bound MthK channel (SI Fig. 17). While the free channel shows the highest intensity for S3, in the toxin-bound sample, S2 shows the highest intensity, suggesting a higher occupancy of this site. S3 and S4 are both similarly intense, which may reflect different configurations with both or only one of them being occupied by an ammonium ion."

In principle, direct polarization (DP) NMR experiments could provide complementary information. However, for the ammonium ion, such experiments are not feasible due to the very low signal

intensities obtained under DP conditions, which would require prohibitively long acquisition times to achieve sufficient signal-to-noise.

Nevertheless, further investigation of the ion dynamics is an important aspect that we aim to address in future studies. To address the question of ion occupancy inside the filter from another view, we additionally performed MD simulations with three instead of 2 ions initially placed inside and confined to the SF. Interestingly, we could observe an unbinding of the toxin in these simulations, likely attributed to the third ion displacing the K27 side chain of CTX inside the S0 binding site of the filter. Once the strongest interaction between MthK and CTX is broken, the toxin is free to detach. These observations provide an explanation of the different intensities in our NMR experiments. Assuming that S2, with the highest intensity, is occupied most of the time, the lower intensities for S3 and S4 could indeed indicate a transient occupation of these two sites, rather than 3 ions being in the SF at the same time. To integrate these new findings, we added the following sentences into the results section of the manuscript (page 15):

> "Interestingly, MD simulations with three ions (S2/S3/S4) initially placed inside the SF resulted in an unbinding of CTX, leading to a decreased K27 presence in S0 (Fig. 5I, Movie S2). This was caused by a repulsion between the side chain of K27 and the additional $K^+$, leading to a detachment of the toxin from the channel. …Overall, MD simulations agree with the ssNMR data and support the predominant occupation of S2 and either S3 or S4, while ions likely exchange between nearby binding sites within the filter."

We also added the following sentence into the discussion part of the manuscript (page 18):

> "This model is supported by MD simulations with three ions in the filter, which result in an unbinding of the toxin. Therefore, two ions inside the SF seems to be the most stable configuration."

4. Related to this, others have measured changes in ion occupancy upon CTX binding in their X-tal/EM data (see the Banerjee 2013 or Sigworth's recent Kv1.2 work). Would be good to discuss briefly.

We have added a small section in the discussion comparing the different ion occupancies upon CTX binding in paddle chimera and Kv1.2 (page 17):

> "These findings align with previous studies showing altered ion occupancies upon CTX binding as in paddle chimera)[26] (S1 vacant; S2, S3, and S4 occupied) and Kv1.2 (offset S1 and S3 occupied; S2 and S4 vacant)[31] by X-Ray or cryo-EM."

5. I am a little surprised that the CTX purification does not include a refolding step to ensure the 3 disulfides are formed correctly. Do the bridges stay intact through the purification? Clearly it works, but just for my curiosity..

Yes, our measurements indicate that the proper fold of CTX is maintained throughout the entire purification process. We regularly tested all protein-containing fractions of the reversed-phase chromatography by MALDI-TOF to identify our target protein. The theoretical mass of CTX is ~4,319 Da. The formation of three disulfide bridges leads to a weight reduction of ~6 Da, while the cyclization of the N-terminal glutamine to pyroglutamate results in a mass loss of ~17 Da. Therefore, properly folded CTX should have a mass of approximately 4,296 Da, which we can detect for our heterologously expressed

wild-type CTX. Additionally, we compared our in-house recombinant CTX with purchased (recombinant) CTX, which shows a mass of ~4313 Da, suggesting the absence of cyclization on the N-terminus. Nevertheless, the fact that our solution NMR measurements produce such clearly resolved spectra with narrow linewidths indicates a unique and stable fold of our CTX preparations. To address this in the manuscript, we added a figure of the MALDI-TOF results in the SI and following sentences in the manuscript (page 4):

> "Vita et al. demonstrated that the native N-terminal pyroglutamate of CTX is not required for proper folding or overall structural stability of the toxin, but the loss of this residue led to a significantly reduced binding affinity[33]. They hypothesized that the N-terminus plays a major role in the specificity of the toxin toward its target. To verify that the heterologously expressed CTX contains three disulfide bridges and an N-terminal pyroglutamate, we performed MALDI-TOF MS on the final purified peptide. Formation of three disulfide bridges results in a mass decrease of 6 Da, and cyclisation of the N-terminal glutamine to pyroglutamate leads to an additional mass reduction of 17 Da. The observed mass of ~4.29 kDa is consistent with these modifications (SI Fig. 3). Noteworthy, formation of the three disulfide bridges seems to be independent from the proper cyclisation of the N-terminal glutamine."

6. The yellow spectrum is a little difficult to see in Fig2a/3a. Is it possible to make it clearer?

We thank the reviewer for the comment, and adjusted the color of the spectrum.

Reviewer #2 (Remarks to the Author):

This manuscript presents a comprehensive structural investigation of the charybdotoxin (CTX)-MthK complex using an integrative approach combining cryo-EM, solution- and solid-state NMR, and molecular dynamics simulations. The technical execution is of high quality, and the combination of multiple complementary methods represents the strength of the work.

Major strengths:
1. The integrative structural biology approach effectively overcomes limitations inherent to any single technique, particularly for characterizing small peptide-protein complexes where symmetry mismatch poses challenges.
2. The solid-state NMR experiments with $^{15}NH_4^+$ ions provide valuable direct evidence for altered ion occupancy in the selectivity filter upon toxin binding, demonstrating occupancy at S2 and S4 while S1 remains vacant.
3. MD simulations reveal important dynamics of the toxin-channel interface, showing that while K27 serves as a stable anchor, other residues exhibit flexibility on the nanosecond timescale.
4. The work provides a well-characterized example of wild-type toxin binding without fusion constructs or heavy-atom derivatives.

We thank the reviewer for their positive and detailed evaluation of our work.

Major concerns:

1. Limited conceptual novelty. While technically sound, the study does not provide fundamentally new mechanistic insights beyond what has been established by previous structures of toxin-channel complexes, particularly the Kv1.2–ChTx structure (Banerjee et al., 2013, eLife) and the recent Kv1.2–dendrotoxin structure (Wu et al., 2024, eLife). The general binding mode—with a conserved lysine inserted into the selectivity filter and peripheral electrostatic/hydrophobic contacts—has been well established. The observation of “structural plasticity” and dynamic binding, while nicely demonstrated here, has also been inferred from previous computational and biophysical studies.

We thank the reviewer for this comment. We agree that the general binding mode of pore-blocking neurotoxins to potassium channels has been established by previous structural studies, including those cited above.

However, we believe that the present work provides additional mechanistic insight that extends beyond these established structural frameworks. In particular, our NMR measurements allow us to directly probe changes in the local chemical environment and conformational dynamics upon toxin binding. The observed chemical shift perturbations suggest that subtle variations in the electrostatic environment, rather than large-scale structural rearrangements, contribute significantly to the high-affinity interaction. Importantly, these observations can be interpreted in the context of the C1 cryo-EM reconstruction of the complex (with the native-like toxin), allowing us to connect subtle NMR shifts with structural features. This aspect, while implicitly present in earlier studies, has not been explicitly characterized at atomic resolution in an experimental setting and may have broader implications for the interpretation of NMR data in similar systems.

Furthermore, our results provide insight into ion occupancy within the selectivity filter in the toxin-bound state. Previous studies describe changes in the ion distribution based on X-Ray or cryo-EM alone, which gives an averaged ion distribution. The combination of ssNMR and cryo-EM in our study allows to further investigate the ion distribution and indicates that only 2 $K^+$ ions are present in the SF when the toxin is bound. This information is not accessible from static structural models alone. Taken together, our study extends existing high-resolution structures by integrating cryo-EM, NMR, and MD simulations to capture both structural and dynamic aspect of the interaction. In particular, the combination of these approaches provides direct experimental insight into electrostatic tuning, conformational dynamics, and ion occupancy changes associated with toxin binding. More broadly, we believe this integrative strategy establishes a valuable framework for future studies of dynamic membrane protein-ligand complexes.

2. Selectivity filter conformation. The authors report that CTX binding does not induce structural rearrangement of the selectivity filter itself, only changes in ion occupancy. This conclusion would benefit from comparison with recent comprehensive analyses of selectivity filter conformations across $K^+$ channels, including during C-type inactivation. For instance, Scherbakov et al. (10.1016/j.ijbiomac.2025.140690) systematically examined SF structures and conformational transitions. Such comparison would strengthen the authors’ interpretation that the filter remains in a “non-collapsed” conformation despite altered ion occupancy.

We thank the reviewer for highlighting this point. Our shown cryo-EM data strongly suggest a non-collapsed filter, as this should lead to visible changes in the electron density map of the C1 reconstruction of the MthK-CTX complex, which we do not observe. To visualize this, we added a comparison of the selectivity filter of the open MthK pore structure (Ye et al., 2010, DOI:

https://doi.org/10.1038/nsmb.1865) and our toxin-bound model. The resulting RMSD value (0.188 Å) and selectivity filter overlay are shown in the SI Figure 5. Additionally, we added the evaluation using the software "siter" described in the mentioned paper (Scherbakov et al., 2025, DOI: 10.1016/j.ijbiomac.2025.140690). The obtained values agree with a conductive selectivity filter state (see SI Figure 5). We added the following sentence to the manuscript (page: 5):

> "Comparison with the published open MthK pore structure (PDB ID: 3LDC)[36] shows a low RMSD value of 0.188 Å for the SF residues and additional evaluation using the software siter[37] further supports the presence of a conductive selectivity filter state in our structure (SI Fig. 5)."

So far, there is no experimental structure of MthK with a collapsed selectivity filter published, which we could have used for comparison.

3. Missed opportunity regarding toxin specificity determinants. The authors identify "structural plasticity" as the basis for CTX's broad channel promiscuity, with K27 as an anchor and flexible peripheral contacts. However, they do not address a critical unresolved question in the field: the M29I mutation in CTX causes a dramatic 1500-fold change in affinity for Kv1.2 (10.1134/S002209302206031X, 10.1016/j.toxicon.2023.107181), yet the structural basis for this specificity switch remains unexplained. The authors identify M29 as showing chemical shift perturbations and suggest it coordinates R34 and Y36, but do not discuss how this position could act as an "evolutionary switch" controlling selectivity across the KTX toxin family. Given the authors' integrated structural approach and focus on binding plasticity, investigating the M29I mutant structure would have provided genuine mechanistic novelty and directly addressed an outstanding question in the field.

We thank the reviewer for mentioning this CTX mutant. While we see peak splitting for M29 in solution NMR and a change when bound to MthK via ssNMR we cannot conclude an interacting partner for M29 on the surface of MthK. As the reviewer describes, different studies show the strong effect of M29 mutations on binding affinity to certain channels (as mentioned above). We can observe in our structure that M29 is "buried" in the toxin, positioned "behind" R34 (CTX) and Y36 (CTX). The increased flexibility of the M29 sidechain compared to isoleucine could explain the behavior observed in previous studies. To account for this, we added the following section in the discussion:

> "Previous studies have demonstrated that mutations at position 29 in CTX can drastically alter binding affinity to different $K^+$ channels. Proposed interaction partners of M29 (CTX) are T449 in Shaker or V381 in Kv1.2 (which corresponds to S66 in MthK, see SI Fig. 6)[51,52]. While methionine is commonly found at this position, some related neurotoxins feature isoleucine[53]. Notably, the CTX mutant M29I results in a ~1500-fold increase in binding affinity for the human Kv1.2 channel, making it more selective to Kv1.2, but leads to a ~1700-fold decrease in blocking activity against Shaker-T449F[51,52]. In our structure, M29 (CTX) is positioned behind R34 (CTX) and Y36 (CTX), suggesting a more indirect or structural role. The increased flexibility of the methionine sidechain compared to isoleucine could explain the previous observations. Methionine can adopt a conformation in which its sidechain is buried within CTX, similar to our structure, allowing the toxin to bind in the presence of bulkier hydrophobic residues near the SF (such as the Shaker-T449F mutant)[51]. The mutation to isoleucine results in a shorter and more rigid sidechain, which favors interactions with smaller hydrophobic residues like V381 in Kv1.2,

> but leads to sterical hinderance with bulkier sidechains as in the Shaker-T449F mutant or Kv1.1 (Y379)[52] . The peak splitting of M29 in our solution NMR data of CTX might represent the "extended" and "buried" state of the residue, however this would need further investigation."

To further investigate the effect of an M29I mutation, we performed MD simulations on the mutated toxin. The M29I mutation did not affect the overall binding of CTX to MthK, with the binding free energy analysis showing the same pattern of interactions for both WT and M29I systems (Fig. 4D and SI Fig. 13). M29 shows interactions with two D64 side chains (MthK), and upon M29I mutation these favorable interactions are conserved. In addition, the binding free energy analysis identified interactions found in cryo-EM as favorable, supporting our experimental data. To account for these new simulations, we added the following paragraph to the results (page: 12):

> "CTX affinity to potassium channels is strongly influenced by a M29I mutation[42–44]. To investigate the effect of this mutation on the binding to MthK we performed additional MD simulations with the CTX M29I mutant. Introduction of this mutant did not change the conformation of the toxin, and the binding mode of CTX M29I to MthK was similar to the MD simulations of CTX WT (SI Fig. 11-12). The mutant toxin remained anchored to the channel with K27 inserted at the S0 site while having a certain degree of rotational flexibility. The overall binding free energy is not influenced by the mutation, with interaction patterns between MthK and CTX being similar for both WT and M29I mutant (SI Fig. 13). Results show that both M29 and M29I residues of CTX conserve favorable interaction with D64 of MthK."

The following sentence was added to the discussion (page 17):

> "MD simulations on the M29I mutant of MthK support our suggestions, showing maintained overall binding free energies in the mutant channel."

Minor points:

• Line 393-400: The comparison with the KcsA-Kv1.3-KTX study is somewhat superficial. The authors suggest differences may be system-dependent but do not explore whether their ammonium-based NMR approach could resolve this question.

We thank the reviewer for this comment and removed this section from the manuscript.

• The cryo-EM resolution (4.1 Å for asymmetric reconstruction) limits atomic-level interpretation of some CTX–MthK contacts. The authors appropriately acknowledge this, but might discuss how future technical advances could improve resolution.

We thank the reviewer for this important point. Further technical developments are expected to enhance the resolution of such complexes. In particular, advances in detector technology, image processing algorithms, and particle classification are likely to facilitate higher-resolution asymmetric reconstructions. We anticipate that continued improvements in image processing software to better address sample heterogeneity will be especially important in the near future. We added the following sentence to the discussion (page 16):

"In this context, continued development of image processing software to better account for conformational sample heterogeneity will likely be critical for resolving such flexible binding partners at higher resolution."

Recommendation:
This is competent structural work with solid technical execution. However, given that the fundamental binding mechanism has been established by previous structures and that the study does not address key unresolved questions (such as the M29I specificity determinant), the conceptual advance appears incremental rather than substantial. The work would be well-suited for a more specialized venue such as Communications Biology, where it would make a valuable contribution to the structural toxinology literature without requiring the broad impact expected for Nature Communications.

We believe that the revised version of our manuscript will appeal to the broad readership of *Nature Communications*. In particular, the newly added simulation data on the M29I mutant, together with the aspects addressed in the answers to the first major concern, further strengthen the manuscript and, in our view, support its suitability for publication in a journal such as *Nature Communications*.

**Reply to reviewer comments:**

Reviewer #1

The authors have sufficiently addressed my minor comments and improved the manuscript. I support publication in the current form.

We thank the reviewer for their positive evaluation of our work.

Reviewer #2

The authors have substantially addressed all major concerns raised in the previous review. The addition of RMSD comparison and siter-based analysis of the selectivity filter conformation (Concern 2) is quantitatively convincing (RMSD = 0.188 Å vs. open MthK, conductive state confirmed). The new MD simulations of the M29I mutant (Concern 3) represent a genuine experimental addition rather than a textual revision, and the proposed structural rationale for methionine flexibility as an "affinity switch" is mechanistically plausible. All minor points have been resolved, including removal of the superficial KcsA-Kv1.3-KTX comparison and addition of a discussion of future cryo-EM resolution improvements.

One point remains partially open: the MD simulations of M29I on MthK show no change in binding free energy, which is noted but not deeply discussed — likely because MthK is a prokaryotic surrogate and the Kv1.2-specific effect of M29I cannot be fully recapitulated in this system. This limitation should be briefly acknowledged in the Discussion. With this minor clarification, the manuscript is suitable for publication.

We thank the reviewer for the acknowledgement of our additional work to address all concerns. For clarification we added the following sentence to the discussion (page 10):

> “However, given that MthK is a prokaryotic potassium channel, our model cannot fully recapitulate the Kv1.2-specific effect of the M29I mutation.”
